# Long-Lived Memory B-Cell Responses following BCG Vaccination

**DOI:** 10.1371/journal.pone.0051381

**Published:** 2012-12-11

**Authors:** Ismail Sebina, Jacqueline M. Cliff, Steven G. Smith, Sarah Nogaro, Emily L. Webb, Eleanor M. Riley, Hazel M. Dockrell, Alison M. Elliott, Julius C. R. Hafalla, Stephen Cose

**Affiliations:** 1 Department of Immunology and Infection, London School of Hygiene and Tropical Medicine, London, United Kingdom; 2 Department of Infectious Disease Epidemiology, London School of Hygiene and Tropical Medicine, London, United Kingdom; 3 Department of Clinical Research, London School of Hygiene and Tropical Medicine, London, United Kingdom; 4 Co-infections Studies Programme, Medical Research Council/Uganda Virus Research Institute Uganda Research Unit on AIDS, Entebbe, Uganda; Institut Pasteur, France

## Abstract

The role of T-cells in immunity against *Mycobacterium tuberculosis (M. tuberculosis)* infection has been extensively studied, however, that of B-cells still remains comparatively unexplored. In this study, we determined the presence and frequencies of mycobacteria-specific memory B-cells (MBCs) in peripheral blood from clinically healthy, Bacillus Calmette Guerin (BCG) vaccinated (n = 79) and unvaccinated (n = 14) donors. Purified protein derivative (PPD)-specific MBCs were present in most donors (both vaccinated and unvaccinated) but their frequencies were significantly higher in vaccinated than in unvaccinated donors. MBCs specific for other mycobacterial antigens [antigen-85A (Ag85A), antigen-85B (Ag85B), 6 kDalton early secretory antigenic target (ESAT-6) and the 10 kDalton-culture filtrate protein (CFP-10)] were less prevalent than those recognising PPD. Furthermore, PPD-specific MBCs were detected in BCG vaccinated donors without ESAT-6 and CFP-10 specific responses. Together, these results indicate that BCG vaccination induces long-lived MBC responses. Similar patterns of response were seen when we examined mycobacteria-specific antibody and T-cell responses in these donors. Our data show for the first time that BCG vaccination elicits long-lived mycobacteria-specific MBC responses in healthy individuals, suggesting a more substantial role of B-cells in the response to BCG and other mycobacterial infections than previously thought.

## Introduction

Immune responses to intracellular pathogens, including to *Mycobacterium tuberculosis* (*M. tuberculosis*), are generally considered to be mediated by cellular immune mechanisms [1], [2]. Indeed, various T-cell subsets and their cytokines have been shown to be a critical component in the protective response to *M. tuberculosis* [3]–[9]. While the literature on antibodies and B-cell responses during *M. tuberculosis* infection remains sparse, accumulating evidence indicates that they may play important roles in *M. tuberculosis* immunity. For instance, mice lacking polymeric immunoglobulin receptors (pIgR) are highly susceptible to *M. tuberculosis* infection, suggesting a role for IgA in immunity to *M. tuberculosis* infection [10]. Balu and colleagues have also reported that an immunotherapeutic human IgA monoclonal antibody might be protective against *M. tuberculosis* [11]. In addition, B-cell aggregates were prominent in lungs extracted from *M. tuberculosis* infected mice, as well as in the granulomas of *M. tuberculosis* infected guinea pigs [12]–[14], suggesting a potential role for B-cells in the immunopathology of *M. tuberculosis* infection. Recently, it has been reported that B-cells in pleural fluid obtained from patients with tuberculous pleuritis actively respond to *M. tuberculosis*-specific antigens, thereby suggesting a potential role for these cells in the local immune response to *M. tuberculosis* in such patients [15]. Moreover, a more recent study has also reported high *FCγRIB* gene expression in tuberculosis (TB) patients compared to healthy individuals, further suggesting that antibody mediated effector mechanisms may well play an important role in immunity against active *M. tuberculosis* infections [16]. Given this context, a revised view of TB immunology in which the roles of cellular and humoral immunity are not mutually exclusive has been suggested [1], [2], [17]. In this model, B-cells are envisaged to shape immune responses to *M. tuberculosis* through mechanisms such as antigen presentation, cytokine production and antibody dependent cell mediated cytotoxicity, as well as by influencing other intracellular killing mechanisms of leukocytes [1], [2].

The role of B-cells in generating effective immune responses to vaccines is unquestioned; the most effective vaccines (including Tetanus and Diphtheria toxoids) generate protective long-lived humoral immune responses. It is generally accepted that this long-term humoral immunity is a product of both long-lived plasma cells that secrete antibodies and memory B-cells (MBCs) [18],[19]. MBCs rapidly and specifically respond to antigenic re-stimulation, thus contributing to both the short-lived and long-lived plasma cell pool and thereby prolonging the period of high serum antibody levels [20]–[23]. In addition, MBCs may persist for a lifetime, thus contributing to the rapid clearance of pathogens following re-exposure throughout the lifetime of the host [17], [24], [25]. For tuberculosis, the only vaccine currently in use is Bacillus Calmatte Guerin (BCG), a live attenuated form of *Mycobacterium bovis* (*M. bovis*). Although it protects against the most severe forms of infant TB such as tuberculous meningitis, its efficacy against adult pulmonary TB is variable across the world [26]. Although the important role of T-cells in the immune response to TB cannot be questioned, a role for B-cells in mycobacteria specific immunity cannot be ruled out. In this report, we demonstrate that MBC responses are elicited after BCG vaccination, are readily detected in peripheral blood and are long lived, suggesting a role for B-cells in immune responses to BCG.

**Figure 1:**
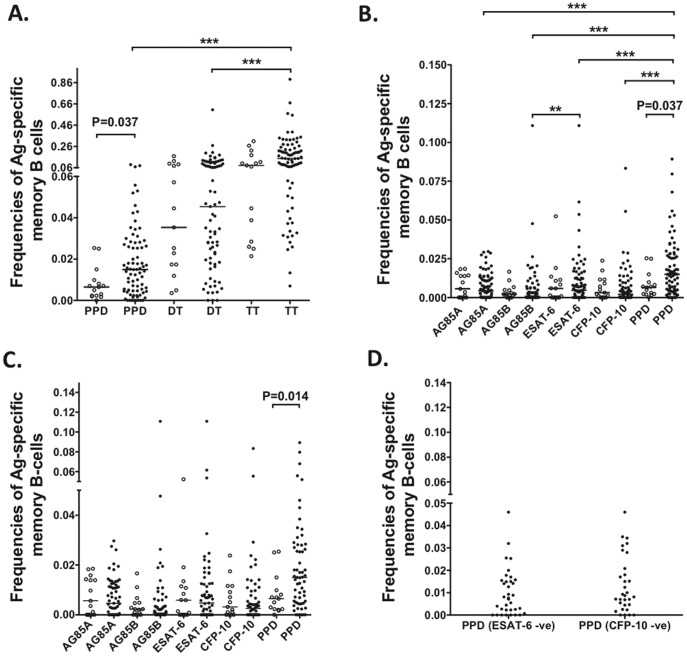
Mycobacteria-specific MBC responses in BCG vaccinated and unvaccinated donors. Frequencies of antigen-specific MBCs were determined by ELISPOT and are presented as a percentage of the total IgG secreting cells. (**A**) and (**B**) Frequencies of mycobacteria-specific MBCs in BCG vaccinated (closed circles) and unvaccinated (open circles) donors, (**C**) MBC responses in vaccinated donors with (closed circles) or without (open circles) any travel history to *M. tuberculosis* endemic areas. (**D**) PPD-specific MBC responses in BCG vaccinated individuals without detectable ESAT-6 or CFP-10 MBC responses. ***, P<0.001; **, P<0.01.

## Materials and Methods

### Study Participants

Clinically healthy BCG vaccinated (n = 79) and unvaccinated (n = 14) adult donors, with a mean age of 25 years participated in this study. Most donors were of United Kingdom (UK)-origin. All were recruited through the London School of Hygiene and Tropical Medicine (LSHTM) anonymous blood donor programme and gave informed written consent to have up to 25 mls of venous blood drawn. The study was approved by the LSHTM ethics committee.

**Figure 2:**
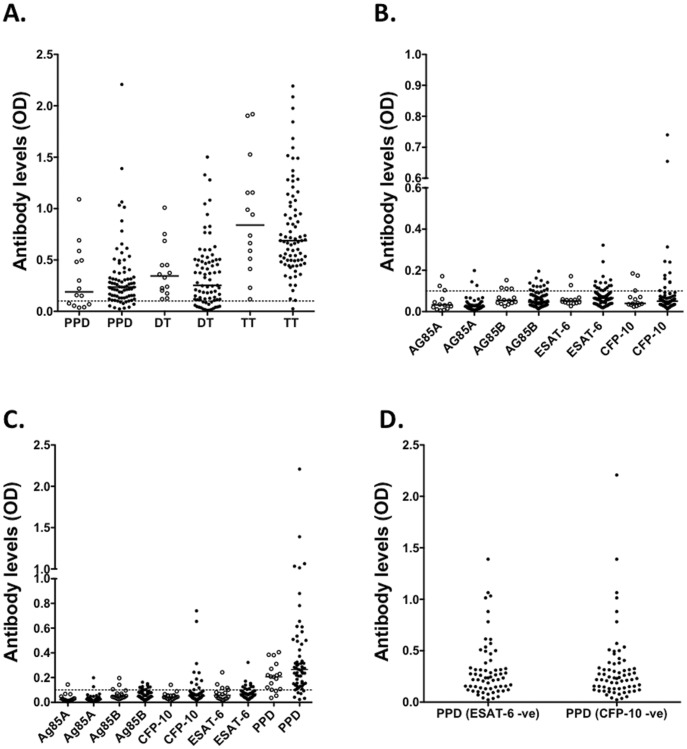
Mycobacteria-specific antibody responses in BCG vaccinated and unvaccinated donors. Antibody responses were determined by ELISA. (**A**) PPD, TT and DT specific antibody levels in BCG vaccinated (closed circles) and unvaccinated donors (open circles). (**B**) Mycobacteria (Ag85A, Ag85B, ESAT-6, CFP-10 and PPD) specific antibody levels in BCG vaccinated (closed circles) and unvaccinated donors (open circles). (**C**) Antibody levels in vaccinated donors with (closed circles) or without (open circles) any travel history to *M. tuberculosis* endemic areas. (**D**) PPD-specific antibody levels in BCG vaccinated individuals without detectable ESAT-6 or CFP-10 antibody responses.

### Antigens and Mitogens

*Mycobacterium tuberculosis* purified protein derivative (PPD) was obtained from Statens Serum Institute (Denmark). ESAT-6 (NR-14868), CFP-10 (NR-14869), Ag85A (NR-14871) and Ag85B (NR-14870) were obtained through BEI Resources (NIAID, USA). Non-adsorbed tetanus toxoid (TT) and diphtheria toxoid (DT) were obtained from the National Institute for Biological Standards and control (NIBSC), UK. CpG-oligonucleotide (ODN-2006, 5′-tcgtcgttttgtcgttttgtcgtt-3′) was obtained through Eurofins MWG/Operon, Germany, while *Phytolacca americana* Pokeweed mitogen (PWM) and *Staphylococcus aureus* Cowan (SAC) were obtained through Sigma-Aldrich, UK.

**Figure 3:**
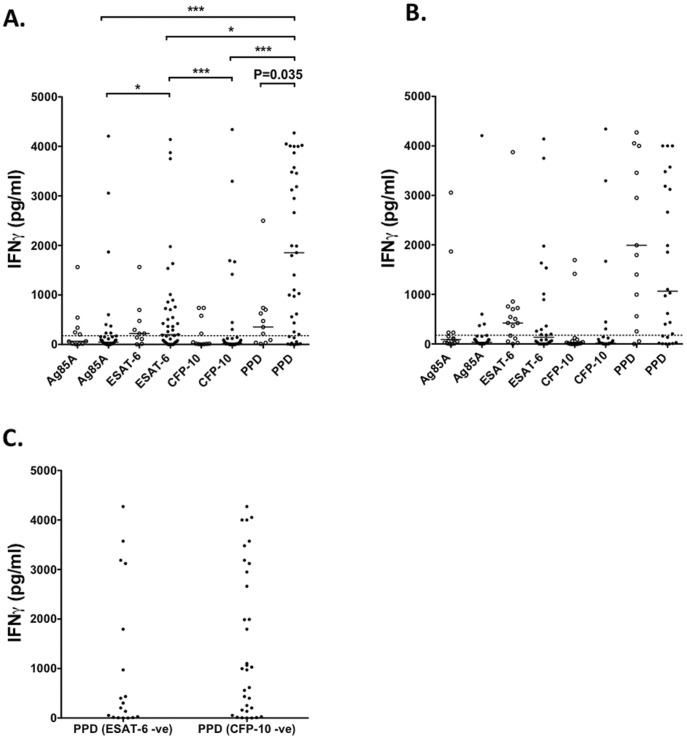
Mycobacteria-specific IFN-γ responses in BCG vaccinated and unvaccinated donors. Responses were measured by stimulating whole blood for 6 days and assaying the culture supernatants for IFN-γ by ELISA. (**A**) Mycobacteria-specific IFN-γ production in BCG vaccinated (closed circles) unvaccinated (open circles) donors. (**B**) IFN-γ responses in BCG vaccinated donors with (closed circles) or without (open circles) any travel history to *M. tuberculosis* endemic areas. (**C**) PPD-specific IFN-γ in BCG vaccinated donors lacking ESAT-6 or CFP-10 responses. *, P<0.05, ***, P<0.001.

### Preparation and Polyclonal Stimulation of PBMCs

Peripheral blood mononuclear cells (PBMCs) were isolated by Ficoll Paque™ Plus (Amersham Pharmacia Biotech, UK) density-gradient centrifugation, washed twice in Hank’s balanced salt solution (HBSS, Invitrogen, UK) and resuspended in RPMI-1640 culture media (Invitrogen, UK) containing 10% Fetal Bovine Serum (FBS), 100 U/ml penicillin, 100 mg/ml streptomycin and 2 mM L-glutamine (all from Sigma-Aldrich, UK). PBMCs (1×10^6^/ml) were added into a 24-well culture plate, with or without a cocktail of 6 µg/ml CpG, 0.5 µg/ml PWM, 1.2 mg/ml SAC and 25 ng/ml recombinant human IL-10 (R&D systems, UK) along with 50 µM β-mercaptoethanol, and stimulated for six days at 37°C in a humidified 5% CO_2_ incubator. IL-10 was added to the polyclonal stimulation cocktail because it increases the differentiation of MBCs into plasmablasts (short-lived plasma cells), thereby increasing the efficiency of the MBC ELISPOT assay [27]–[30].

### ELISPOT

B-cell enzyme-linked immunosorbent spots (ELISPOTs) were performed as described previously [31]. Briefly, 96-well filter plates (Millipore, MAHAS4510) were coated with either 100 µl of PBS containing mycobacterium-specific recombinant proteins (6 µg/ml PPD, 10 µg/ml ESAT-6, Ag85A, Ag85B, and CFP-10), 2 µg/ml TT, 2 µg/ml DT, 10 µg/ml AffiniPure F(ab’)_2_ fragment donkey anti-human IgG (H+L) (Jackson ImmunoResearch) or with PBS alone. Polyclonal stimulated cells (4×10^5^ cells/ml) were transferred directly into each antigen-coated well. Plates were developed with biotin-SP-conjugated AffiniPure fragment donkey anti-human IgG (Jackson ImmunoResearch), strepavidin- AKP (BD biosciences) and its substrate (AP-conjugate substrate kit, Biorad, USA). Developed spots were counted using an AID ELISPOT reader (AID Diagnostika, Germany) and AID ELISPOT software 4.0. The frequencies of antigen-specific MBCs are presented as a percentage of the total IgG secreting cells per million stimulated PBMCs. A positive ELISPOT response was defined as the presence of two or more spots in each replicate well, with the total number of spots in antigen coated wells being at least twice that observed in negative control wells.

### Antibody ELISA

Immulon 4HB plates (Dynatech, USA) were coated with either TT (0.5 µg/ml), DT (0.5 µg/ml), or, 2 µg/ml of PPD, Ag85A, Ag85B, ESAT-6 or CFP-10 in bicarbonate buffer (pH 9.6) overnight at 4°C and then blocked with 1% skimmed milk for 2 hours at room temperature. After washing the plates, 50 µl of diluted plasma samples (1∶2000 for TT and DT and 1∶200 for mycobacterial antigens) was added in duplicate into each well and incubated overnight at 4°C. Plates were then washed and developed with 50 µl of a 1∶5000 diluted anti-human IgG horseradish peroxidase conjugate (Caltag Laboratories, Invitrogen, Paisley, UK) and *O*-phenylenediamine (OPD) substrate (Sigma). The enzyme reaction was terminated with 30% w/v sulphuric acid and absorbance read at 492 nm on an ELISA plate reader (Dynex Technology-MRX II). Corrected OD values (where average background (0.08) was subtracted) were used in subsequent analysis. The average OD value (0.1) of a negative control plasma sample (from a BCG naïve infant) was used as an arbitrary cutoff in determining positive and negative responses.

### Whole Blood Assay

Whole blood assay (WBA) for mycobacteria*-*specific interferon gamma (IFN-γ) responses was performed as previously described [32], [33]. Briefly, heparinised blood was diluted to a final concentration of 1 in 4 with serum-free medium (RPMI supplemented with L-glutamine, penicillin and streptomycin), and 200 µl per well added to 96-well, round-bottomed plates (TC Microwell, NUNC A/S, Ros-kelde, Denmark). Blood was stimulated with or without 10 µg/ml ESAT-6, CFP-10, Ag85A, PPD or phytohaemagglutinin (PHA, Sigma, UK), incubated for six days at 37°C in a humidified 5% CO_2_ incubator, and the supernatants then harvested and stored at −80°C. IFN-γ was measured in supernatants using OptEIA ELISA Kits (BD Pharmingen, USA) as per the manufacturers guidelines. Background (concentration in unstimulated wells) was subtracted from IFN-γ concentrations in antigen-stimulated wells to obtain antigen-specific responses. A cutoff of 125 pg/ml was set to identify positive from negative responses.

### Statistical Analysis

Statistical analysis was performed using Stata 10.1. Frequency distributions of antigen specific MBC, antibody and IFN-γ responses were highly skewed, and transformation did not normalize the data, therefore non-parametric statistical tests were used throughout. Frequencies of MBC, antibody and of IFN-γ responses were compared between all antigens using Friedman’s and Kruskal Wallis tests for the overall comparison followed by Dunn’s post-test for each individual pairwise comparison. The distribution of frequencies for each response was compared between BCG vaccinated and unvaccinated donors, and between donors with and without a history of travel to *M. tuberculosis* endemic areas, using the Kruskal Wallis test.

## Results and Discussion

### Mycobacteria-specific Memory B-cells in BCG Vaccinated and Unvaccinated Donors

Mycobacteria-specific MBC responses have not been previously identified in healthy BCG vaccinated individuals, and our initial studies aimed to investigate whether we could detect such responses. We collected PBMCs from adult BCG vaccinated (n = 79) and BCG unvaccinated (n = 14) healthy donors residing in the UK and performed mycobacteria-specific B-cell ELISPOTs on these PBMCs. PPD-specific MBCs were detected in both vaccinated and unvaccinated individuals, however their frequencies were significantly higher in vaccinated individuals than in their unvaccinated counterparts (P<0.037, Figure 1A). As expected, Tetanus Toxoid (TT) and Diphtheria Toxoid (DT), which served as positive controls for two effective antibody-inducing vaccines, stimulated strong MBC responses and were unaffected by BCG vaccination (Figure 1A).

Having detected PPD-specific responses in these donors, we next examined whether they had MBC responses to other mycobacterial antigens. Along with PPD, we could detect responses to Ag85A, Ag85B, ESAT-6 and CFP-10 in both vaccinated and unvaccinated donors (Figure 1B). In the vaccinated donors, frequencies of PPD-specific MBCs were significantly higher than those specific for any other antigen (P<0.001), however there were no differences between responses to these antigens in the unvaccinated individuals (Figure 1B). Some BCG vaccinated individuals may have travelled to *M. tuberculosis* endemic countries, and therefore we could not rule out the possibility of other mycobacterial exposures contributing to the mycobacteria-specific MBC responses in this group. To address this, we compared responses in vaccinated individuals who had or had not travelled, lived or worked in *M. tuberculosis* endemic areas during their lifetime (Figure 1C). Frequencies of PPD-specific MBCs were significantly higher in vaccinated individuals who had travelled to mycobacteria endemic areas compared to those who had not (P = 0.014), implying that some BCG-vaccinated donors may well have been exposed to mycobacterial species other than BCG. There were no significant differences in responses to other mycobacterial antigens tested. To confirm whether BCG vaccination could independently induce MBC responses, we investigated whether PPD-specific MBCs were present in individuals lacking either ESAT-6 or CFP-10 responses. Since these proteins are missing in BCG, we hypothesized that PPD-specific responses in BCG vaccinated individuals that lack these responses are due to the vaccine itself, rather than some other exposure. In agreement with this hypothesis, we were able to detect PPD-specific MBCs in BCG vaccinated individuals that lacked responses to ESAT-6 or CFP-10 (Figure 1D). Although there were no significant differences in PPD-specific MBC responses in these individuals compared to those in the unvaccinated donors, we noted that the majority of the BCG unvaccinated donors had travelled, lived or worked in mycobacteria endemic areas and thus could have been exposed to mycobacteria, which may have contributed to the responses detected in these unvaccinated individuals. Of note, unvaccinated donors without any travel history to endemic areas had PPD-specific MBC responses that were lower than those detected in BCG vaccinated individuals lacking ESAT-6 and CFP-10 specific MBCs (data not shown). Further statistical analysis was limited by the low numbers of samples, however these observations do suggest that the majority of the MBC responses detected in BCG vaccinated donors lacking ESAT-6 and CFP-10 responses are likely due to BCG vaccination, rather than previous exposure to other mycobacterial species. Of note, the demographic data we collected from our BCG vaccinated donors showed that the minimum time from BCG vaccination to enrollment was 13 years, while the maximum time from vaccination was 45 years (data not shown). Stratification by age had no effect on the frequencies of MBCs detected in these donors. Together, these findings indicate that BCG vaccination itself elicits long-lived MBC responses.

### Mycobacteria-specific Antibody Responses in BCG Vaccinated and Unvaccinated Donors

Both plasma cells and MBCs contribute to the production of long-lived antibody responses. We therefore investigated whether antibody responses were also detectable in these healthy donors. We compared mycobacteria-specific antibody levels in the plasma of BCG vaccinated (n = 79) and BCG unvaccinated donors (n = 14). Unlike the MBC responses, mycobacteria-specific antibody levels were similar between the two sets of donors, both for PPD (Figure 2A) and other mycobacterial antigens (Figure 2B). In addition, there were no differences in mycobacteria-specific antibody levels between individuals with or without travel history to *M. tuberculosis* endemic areas (Figure 2C). These data, and the fact that we were able to detect mycobacteria-specific antibodies in individuals without detectable antibodies to ESAT-6 or CFP-10 (Figure 2D), imply that unvaccinated individuals may possess cross-reactive antibodies and MBCs, induced by exposure to other pathogens or to non-tuberculous mycobacteria or following BCG vaccination.

### Mycobacteria-specific IFN-γ Responses in BCG Vaccinated and Unvaccinated Donors

To determine whether T-cell specific responses were also present in these healthy individuals, we measured mycobacteria-specific IFN-γ in culture supernatants from BCG vaccinated (n = 43) and unvaccinated (n = 11) donors. PPD-specific IFN-γ responses were significantly higher in vaccinated individuals compared to their unvaccinated counterparts (P = 0.035), showing that BCG vaccination elicits robust T-cell responses (Figure 3A). In BCG-vaccinated individuals, PPD-specific IFN-γ responses were more prevalent than those specific for ESAT-6 (P<0.05), Ag85A (P = 0.001), and CFP-10 (P<0.001). There were no differences in mycobacteria-specific IFN-γ responses between BCG-vaccinated individuals with or without any travel history to mycobacteria endemic areas (Figure 3B). This suggested, as with the MBC data (Figure 1C), that the T-cell responses in vaccinated individuals lacking a travel history to *M. tuberculosis* endemic areas was due to vaccination itself, rather than exposure to other mycobacteria. To further confirm this, we investigated whether vaccinated individuals lacking ESAT-6 or CFP-10 responses gave detectable IFN-γ responses following PPD stimulation (Figure 3C). We found that these individuals did, in fact, give good responses to PPD, showing that BCG itself also elicits long-lived memory T-cell responses. These data extend the work of Dockrell and colleagues [26], [34], who showed positive T-cell responses to BCG in vaccinated infants and adolescents living in the UK.

### Concluding Remarks

This study shows the presence of long-lived humoral and cellular responses specific to certain mycobacterial antigens in peripheral blood. In particular, this is the first study to report the presence of detectable numbers of mycobacteria-specific MBCs in peripheral blood from healthy individuals living in low endemic areas for tuberculosis, and that these responses last for up to 45 years following BCG vaccination. These findings may provide a new avenue to investigate possible biomarkers of latent and active *M. tuberculosis* infections. We further postulate that differences in humoral immune responses may reflect the clinical status of *M. tuberculosis* infected individuals, as suggested by others [35]–[37]. We specifically hypothesize that the presence of antigen-specific plasmablasts within the peripheral circulation may reflect on-going infection (active) whereas antigen-specific MBCs in the absence of plasmablasts may reflect a resolved or subclinical infection.
